# The Long-Term Consequences of Early Life Exposure to Tsunami and Conflict on Adolescents in Sri Lanka

**DOI:** 10.1177/10105395231151730

**Published:** 2023-01-25

**Authors:** Delan Devakumar, Laura Busert, Manoji Gitanjali Sathiadas, Pushpika Jayawardana, Angela Arulpragasam, Clive Osmond, Caroline H. D. Fall, Jonathan C. K. Wells, V. Pujitha Wickramasinghe

**Affiliations:** 1 Institute for Global Health, University College London, London, UK; 2 Great Ormond Street Institute of Child Health, University College London, London, UK; 3 Faculty of Medicine, University of Jaffna, Jaffna, Sri Lanka; 4 Faculty of Medicine, University of Ruhuna, Galle, Sri Lanka; 5 Faculty of Health Care Sciences, Eastern University, Sri Lanka, Batticaloa, Sri Lanka; 6 MRC Lifecourse Epidemiology Centre, Southampton General Hospital, University of Southampton, Southampton, UK; 7 Faculty of Medicine, University of Colombo, Colombo, Sri Lanka

**Keywords:** tsunami, conflict, war, adolescent, nutrition, anthropometry

## Abstract

The consequences for adolescent health due to early life exposure to natural disasters combined with war are not known. We collected data from adolescents aged 12-13 years in Sri Lanka whose mothers were pregnant during the Indian Ocean tsunami in 2004 in a tsunami-affected region (n = 22), conflict-affected region (n = 35), conflict-plus-tsunami-affected region (n = 29), or controls in areas unaffected by either (n = 24). Adjusted body mass index (BMI)-for-age z-scores were 1.3, 1.0 and 2.0 for conflict, tsunami, and conflict-plus-tsunami, respectively, compared with the control group. Greater skinfold thickness and higher diastolic blood pressure were found in adolescents born in the conflict zone but no differences were found in height, head circumference, and waist circumference, or blood results, with the exception of serum insulin. Being born after a natural disaster or during conflict was associated with increased BMI and body fat during adolescent, which are associated with longer-term risk of noncommunicable disease.

## What We Already Know

- Natural disasters and conflicts can lead to immediate adverse health consequences in the population.
- The evidence on early life stress shows long-term negative consequences for health.

## What This Article Adds

- Early life exposure to disaster combined with exposure to war may lead to changes in anthropometry and blood pressure in young adolescents in Sri Lanka.
- Adolescents born during the Sri Lanka civil war were more likely to be heavier, with increased fat. This was especially so, if there were born in areas affected by the tsunami.

## Introduction

Natural disasters and wars are known to have devastating effects on child health in the short-term, but it is often assumed that once they are over, the population is no longer at risk.^1-3^ Evidence is emerging, mostly from high-income countries, on the effect of disasters on subsequent health outcomes, showing for example that the exposure of pregnant women to these extreme events can lead to high levels of maternal stress/mental illness, malnutrition, injury, and infection, that can further result in poorer health outcomes in their children.^4,3^ Given their enormity, the combination of disasters caused by natural events and war are likely to have profound lasting effects on adolescents, however the importance of early life exposure to these stresses has not been assessed.

There is now compelling evidence supporting the hypothesis that environmental influences impacting mothers during pregnancy can shape health responses in the offspring decades later.^5^ Relevant exposures include maternal nutritional status and dietary intake prior to and during pregnancy, maternal infection, and maternal psychosocial stress.^6^ Among the outcomes in the offspring showing associations with these exposures are markers of growth and nutritional status, cardiometabolic risk, and mental health.^5^ Given that the capacity for plasticity through epigenetic change has itself been shaped by selection, we assume that developmental adjustments broadly promote survival and reproductive fitness, though potentially at a cost to other outcomes such as health and longevity.^7^ To understand these associations, we draw on evolutionary life history theory, which assumes that every organism is under selective pressure to allocate energy and resources across four competing functions. The four relevant functions are maintenance (effectively, homeostasis), growth, reproduction, and defense against pathogens and predators.^7^ Since energy and resources are finite, this gives rise to trade-offs between the functions. One of the key predictions of life history theory is that, as environments become harsher or more dangerous and life expectancy falls, the returns on investment in maintenance and growth decline, since the long-term benefits may never materialize.^8^ Instead, selection favors increased allocation of resources to survival (defense) and reproduction, as demonstrated for adolescent daughters exposed to low maternal nutritional investment,^9,10^ though findings were less consistent in sons, potentially as males have different mechanisms for investing in reproduction.^11^

We therefore hypothesized that early-life exposure to maternal trauma would induce trade-offs favoring survival and reproduction over growth and maintenance, and that the consequences would be greatest in those born in the areas exposed to both conflict and tsunami. Specifically, we expected that the offspring of mothers exposed to trauma during pregnancy would have shorter height and greater body fat with a more central distribution. Height is a marker of linear growth and also has implications for maintenance, being correlated with the size of the vital organs,^12^ while body fat is a marker of energy reserves for immune function, and reproduction in females.^4^ Conversely, we expected little difference between the groups in head circumference, as the brain is considered to be the part of the body most buffered against stresses during development.^13^

## Methods

The following locations in the country were chosen based on the exposure to the relevant stress condition: Galle (tsunami), Kilinochi and Manmunai (conflict), Mullativu and Koralai Pattu (conflict-plus-tsunami), and Akmeemana (control; minimally affected by either tsunami or war). Sri Lanka provides a rare opportunity to investigate long-term effects of both a major natural disaster (Indian Ocean tsunami, 2004) and conflict (1983-2009). Both led to large-scale death and destruction. The regions of the country affected by the war and the tsunami were well demarcated, allowing us to compare families who live relatively close to each other but who had different exposures to the war and tsunami. However, our use of the term “control” does not imply that the people in these areas were completely unaffected by the war or tsunami, simply that they were not directly affected by the fighting or destruction. A control site was chosen as close as possible to the tsunami site to make the population samples as similar as otherwise possible and also for pragmatic reasons to allow one study team to access both sites. It would not have been possible to choose a control site close to the conflict sites.

An adolescent was eligible for inclusion if his / her mother was pregnant at any point during the tsunami on the 26 December 2004. After obtaining approval from educational authorities, schools occupied by children of the relevant age were identified and based on date of birth, potential eligible children were identified. Then, the residence of their mothers at the time of pregnancy were identified and if within the designated localities, these children were recruited. It was assumed that they were exposed to the war, by the locations that they lived in. Informed consent was taken from the parent or guardian in their own language and assent was taken from the adolescent. Interviews were conducted with the mothers including a questionnaire on socioeconomic status (components including 10 components that would make a household poor, organized into three domains: education; health; and living standard). The questionnaire tools were translated and blind back-translated into Sinhalese and Tamil.

The main outcomes were height, weight, and height-for-age and body mass index (BMI)-for-age *z* scores according to the World Health Organization (WHO) Child Growth Standards for children aged 5 to 19 years,^14^ fat mass distribution based on skinfold thickness, body circumferences, and blood pressure. These data were collected by five data collectors, who were trained prior to the study. Anthropometry was measured following University College London (UCL) Great Ormond Street Institute of Child Health guidelines (based on an Anthropometric Standardization Reference Manual).^15^ Duplicate measures of standing height were made using a Leicester stadiometer (Seca 213, Hamburg, Germany), accurate to 1 mm. Weight was measured with a Seca 813 digital weighing scales (Hamburg, Germany), accurate to 100 g. Body circumferences were measured in duplicate using Seca 201 tapes (Hamburg, Germany), accurate to 1 mm, the adolescent standing in the appropriate anatomical position. Left biceps, triceps, subscapular and supra-iliac skinfold thicknesses were measured in triplicate with a Harpenden caliper (Assist Creative Resource, Wrexham, UK), accurate to 2 mm.

We used an electronic blood pressure monitor (Omron Healthcare Ltd, Japan) with a pediatric or adult cuff as required.^16^ Two readings were taken and the lowest value was recorded. Pubertal stage was assessed by a self-assessment. After consent from the parent/care giver, descriptions of the pubertal stages (Tanner stages) were given to the child and the child would confidentially choose the number most appropriate to them.

Venous blood samples were taken using aseptic techniques by the data collectors, who were already trained in taking blood from children. Full blood count, fasting glucose, and HBA1C were tested in the laboratory in the local teaching hospital and frozen samples were transported to the University of Colombo in batches where they were tested for lipids, insulin, and cortisol levels. Details of the blood sample testing are given in the appendix.

Ethics approval was granted by the University of Colombo (EC-17-074) and University College London (2744/003) Research Ethics committees. Participants/parents could withdraw from the study or remove their data at any point, but none chose to do so. To ensure the safety of our participants, any adverse events or abnormal test results were reported to the research leads at the site, all of whom are consultant pediatricians.

### Life History Conceptual Model

**Figure 1.**
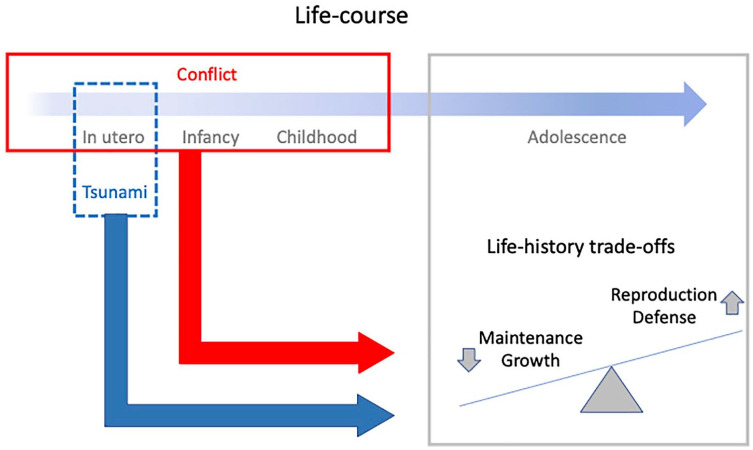
Schematic diagram illustrating our conceptual framework. Exposure in utero to the tsunami, or in early in life to conflict, are both expected to elicit life history trade-offs favoring survival (defense) and reproduction over maintenance and growth. These trade-offs may be more extreme among those who experienced both stresses.

Differentiating life history functions in physical traits is not easy, as there is not a direct one-to-one mapping. We treated height, weight, and head circumference as the primary markers of investment in growth; BMI, circumferences of the arm, hip, thigh and calf, and biceps and triceps skinfolds as markers of investment in reproduction; waist circumference and subscapular and suprailiac skinfolds as markers of investment in defense; and blood pressure and blood biochemistry as markers of investment in maintenance (maintaining metabolic health) (Figure 1).

### Data Management

Data were entered into Microsoft Excel (Microsoft Corp, USA). No analyses or shared data included the names of participants or families. Questionnaires were stored in a locked office and remained at the research site.

### Statistical Analysis

Analysis was carried out in Excel (Microsoft Corp, USA) and Stata (15.1; Stata Corp, USA). Summary statistics were produced for the overall sample and in each *in-utero* exposure group. The WHO Child Growth Standards for children aged 5-19 were applied to generate *z* scores for height- and body mass index (BMI) for age.^14^ The socioeconomic data were used to calculate the Mulitidimensional Poverty Index (MPI) for an individual household and a region. A higher score indicates being multidimensionally poorer. Individual household MPI scores were used in the regression analyses, regional scores reported in Table 1 taking into account “the proportion of people who experience multiple deprivations and the ‘intensity’ of their deprivation.”^17^ We first compared each exposure group separately against controls (using location as a categorical variable) using univariable regression models. Outcome variables were anthropometry variables, body composition (skinfold thickness), blood pressure, and blood test results, as specified in Tables 1 and 2. We constructed a conceptual diagram and chose confounding variables from this (see Appendix). Correlation coefficients were calculated for each confounding variable with each outcome (Appendix, Table 1). The distribution of the individual MPI variable showed a right skew, so was transformed by taking its square root. Adjusted analysis was then performed, again comparing each location separately against the controls. All models controlled for age, gender, socioeconomic status (square root of MPI), and maternal height, except anthropometry *z* scores (already adjusted for age and gender) which were adjusted for socioeconomic status and maternal height only. Correlation coefficients for paternal height did not indicate a relationship with the outcomes (Appendix, Table 1). Multiple testing correction was performed using the Benjamini-Hochberg method (Appendix, Table 2).^18^ This test gives a *P* value cut-off taking into consideration the number of analyses run. We performed 24 analyses, with three comparisons per outcome (72 outcomes in total). If applying a *P* value “cut-off” of .05 initially, the Benjamini-Hochberg test gives an adjusted *P* value of .01.

**Table 1.** Characteristics of Participants and Summary of Main Outcomes by Location Where Participant Was Born.

	Overall	Control	Conflict	Tsunami	Conflict plus tsunami
N	110	24	35	22	29
Mean age (years) (SD)	13.2 (0.3)	13.2 (0.2)	13.3 (0.4)	13.0 (0.3)	13.1 (0.2)
Gender (girls)	46	9	14	9	14
Pubertal stage (mean)^a^ (SD)	1.8 (0.8) n = 109	1.3 (0.5) n = 24	2.4 (0.7) n = 34	1 (0) n = 22	2.2 (0.7) n = 29
Multidimensional index score for the region	0.113	0.024	0.185	0	0.191
Parental height					
Maternal height (cm) (SD)	156.0 (6.7) n = 95	154.7 (7.3) n = 23	153.4 (7.1) n = 25	158.6 (6.9) n = 22	157.5 (4.1) n = 25
Paternal height (cm) (SD)	165.8 (6.8) n = 92	165.1 (7.3) n = 24	163.6 (7.7) n = 22	169.2 (4.3) n = 22	165.3 (6.5) n = 25
Anthropometry					
Height (cm) (SD)	149.7 (7.7)	153.4 (7.9)	147.8 (8.1)	150.0 (4.8)	148.6 (8.2)
Weight (kg) (SD)	38.0 (8.9)	39.0 (7.3)	36.3 (9.2)	39.8 (9.8)	37.8 (9.1)
Height for age *z* score	−0.95	−0.56	−1.26	−0.78	−1.03
BMI for age *z* score	−1.18	−1.25	−1.41	−0.84	−1.09
Head circumference (cm) (SD)	52.4 (1.6)	52.5 (1.5)	52.3 (1.6)	52.6 (2.0)	52.2 (1.6)
Mid-upper arm circumference (cm) (SD)	21.5 (3.2)	21.7 (2.5)	20.8 (3.0)	21.9 (4.3)	21.7 (2.9)
Waist circumference (cm) (SD)	64.1 (7.9)	69.4 (5.0)	60.6 (7.5)	64.0 (7.1)	64.0 (8.6)
Hip circumference (cm) (SD)	72.5 (8.9)	71.6 (6.7)	69.4 (7.9)	75.1 (11.0)	75.1 (9.1)
Thigh circumference (cm) (SD)	40.7 (5.9)	43.6 (4.3)	37.7 (5.1)	43.0 (6.6)	40.2 (5.6)
Calf circumference (cm) (SD)	26.3 (4.8)	20.5 (2.6)	27.1 (3.2)	28.8 (4.4)	28.4 (4.1)
Skinfold thickness					
Biceps (mm) (SD)	6.7 (4.2)	5.4 (2.3)	6.5 (3.9)	5.2 (2.7)	9.3 (5.6)
Triceps (mm) (SD)	10.0 (4.6)	8.6 (3.0)	9.8 (4.6)	8.3 (3.0)	12.6 (5.6)
Subscapular (mm) (SD)	9.0 (4.6)	8.3 (2.6)	9.2 (5.3)	6.5 (2.5)	11.3 (5.0)
Suprailiac (mm) (SD)	11.5 (6.0)	9.8 (4.0)	12.7 (6.4)	7.8 (3.7)	14.4 (6.6)
Blood pressure					
Systolic (mmHg) (SD)	101.0 (10.6)	100.5 (10.6)	104.1 (10.2)	99.2 (11.3)	99.2 (10.4)
Diastolic (mmHg) (SD)	66.4 (7.4)	63.2 (5.6)	67.4 (6.6)	65.9 (8.2)	68.0 (8.3)
Blood results					
Fasting glucose (mg/dL) (SD)	90.4 (9.6)	94.3 (9.7)	90.1 (10.5)	94.1 (8.2)	84.9 (6.4)
HBA1C (%) (SD)	5.3 (0.5)	5.5 (0.7)	5.2 (0.3)	5.6 (0.6)	5.1 (0.2)
Serum insulin mIU/L (SD)	7.1 (6.0)	5.0 (3.4)	8.6 (7.0)	7.0 (4.0)	7.1 (7.1)
Cortisol mcg/dL (SD)	5.7 (2.7)	6.3 (2.9)	5.2 (2.3)	6.9 (3.6)	5.0 (1.8)
Cholesterol mg/dL (SD)	158.6 (29.0)	160.9 (30.8)	162.4 (33.5)	157.0 (30.4)	153.5 (19.9)
Triglycerides mg/dL (SD)	93.2 (49.4)	80.7 (25.4)	104.9 (65.1)	82.0 (43.1)	97.8 (44.8)
High-density lipoprotein mg/dL (SD)	36.1 (11.7)	38.3 (8.6)	38.3 (13.1)	35.0 (11.2)	32.3 (11.9)
Low-density lipoprotein mg/dL (SD)	99.6 (23.9)	97.0 (24.7)	100.0 (21.6)	97.6 (26.7)	102.9 (24.5)

Abbreviations: BMI, body mass index; SD, standard deviation.

a Tanner stage, represented as an average.

**Table 2.** Unadjusted and Adjusted Models Showing the Association (Regression Coefficients and 95% Confidence Intervals) Between Location of Birth and Adolescent Nutrition and Blood Pressure Outcomes Compared With the Baseline (Control Region).

	Unadjusted models^a^			Adjusted models^a,b^		
	Conflict [95% CI]	Tsunami [95% CI]	Conflict plus tsunami [95% CI]	Conflict [95% CI]	Tsunami [95% CI]	Conflict plus tsunami [95% CI]
Height (cm)	**−5.6 [−9.6, −1.7]**	−3.5 [−7.9, 1.0]	−**4.8 [**−**8.9**, −**0.6]**	0.7 [−4.0, 5.5]	−0.1 [−4.5, 4.4]	1.2 [−3.6, 6.0]
Weight (kg)	−2.68 [−7.36, 1.99]	0.88 [−4.32, 6.09]	−1.17 [−6.03, 3.70]	**6.85 [1.45, 12.25]**	**5.95 [0.93, 10.97]**	**9.30 [3.82, 14.78]**
Height for age	−**0.7 [**−**1.2**, −**0.2]**	−0.3 [−0.8, 0.4]	−0.5 [−1.0, 0.1]	0.1 [−0.5, 0.8]	0.0 [−0.6, 0.6]	0.2 [−0.4, 0.8]
BMI for age	−0.2 [−0.1, 0.7]	0.4 [−0.5, 1.3]	0.2 [−0.7, 1.0]	**1.3 [0.4, 2.2]**	**1.0 [0.2, 1.9]**	**2.0 [1.1, 2.9]**
Head circumference (cm)	−0.2 [−1.1, 0.6]	0.1 [−0.9, 1.0]	−0.3 [−1.2, 0.6]	0.3 [−0.9, 1.5]	0.4 [−0.7, 1.5]	0.3 [−0.9, 1.5]
Mid-upper arm circumference (cm)	−0.8 [−2.5, 0.8]	0.3 [−1.6, 2.1]	0.1 [−1.7, 1.8]	**2.1 [0.1, 4.0]**	**1.9 [0.1, 3.7]**	**3.4 [1.5, 5.4]**
Waist circumference (cm)	−**8.8 [**−**12.6**, −**5.0]**	−**5.4 [**−**9.6**, −**1.1]**	−**5.4 [**−**9.4**, −**1.4]**	−4.4 [−9.3, 0.5]	−2.7 [−7.3, 1.8]	0.1 [−4.9, 5.0]
Hip circumference (cm)	−2.2 [−6.7, 2.4]	3.5 [−1.6, 8.6]	3.5 [−1.2, 8.3]	4.3 [−1.4, 9.9]	5.1 [−0.2, 10.3]	**9.3 [3.5, 15.0]**
Thigh circumference (cm)	−**5.9 [**−**8.7**, −**3.0]**	−0.6 [−3.8, 2.6]	−**3.4 [**−**6.3**, −**0.4]**	−0.9 [−4.4, 2.5]	1.9 [−1.3, 4.0]	2.0 [−1.5, 5.5]
Calf circumference (cm)	**6.7 [4.8, 8.6]**	**8.4 [6.2, 10.5]**	**7.9 [5.9, 9.9]**	**7.9 [5.3, 10.5]**	**9.3 [6.9, 11.7]**	**10.0 [7.4, 12.6]**
Skinfold thickness						
Biceps (mm)	1.1 [−1.0, 3.2]	−0.1 [−2.5, 2.2]	**3.9 [1.7, 6.1]**	**3.5 [0.9, 6.3]**	0.5 [−2.0, 3.1]	**6.0 [3.2, 8.7]**
Triceps (mm)	1.2 [−1.1, 3.5]	−0.3 [−2.9, 2.2]	**4.0 [1.6, 6.4]**	**3.1 [0.3, 5.9]**	0.2 [−2.4, 2.9]	**5.7 [2.9, 8.5]**
Subscapular (mm)	1.0 [−1.3, 3.2]	−1.8 [−4.3, 0.7]	**3.0 [0.7, 5.4]**	**3.6 [0.9, 6.4]**	−1.0 [−3.6, 1.5]	**5.2 [2.4, 8.0]**
Suprailiac (mm)	**3.0 [0.0, 5.9]**	−2.0 [−5.2, 1.3]	**4.6 [1.6, 7.7]**	**5.1 [1.7, 8.6]**	−1.5 [−4.7, 1.8]	**7.3 [3.8, 10.8]**
Blood pressure						
Systolic blood pressure (mmHg)	3.6 [−2.0, 9.1]	−1.4 [−7.5, 4.8]	−1.3 [−7.1, 4.5]	6.3 [−1.4, 13.9]	1.7 [−5.4, 8.8]	3.2 [−4.6, 10.9]
Diastolic blood pressure (mmHg)	**4.3 [0.5, 8.1]**	2.8 [−1.5, 7.0]	**4.8 [0.9, 8.8]**	**5.6 [0.5, 10.7]**	4.2 [−0.5, 9.0]	**6.7 [1.5, 11.8]**
Blood results						
Fasting glucose (mg/dL)	−4.2 [−8.9, 0.4]	−0.2 [−5.4, 5.0]	−**9.4 [**−**14.2**, −**4.5]**	−5.2 [−10.9, 0.5]	3.6 [−1.7, 8.9]	−5.3 [−11.1, 0.4]
HBA1C (%)	−**0.3 [**−**0.5**, −**0.0]**	0.1 [−0.1, 0.4]	−**0.4 [**−**0.6**, −**0.1]**	−0.3 [−0.6, 0.1]	0.1 [−0.2, 0.5]	−0.3 [−0.7, 0.0]
Serum insulin (mIU/L)	**3.6 [0.3, 6.8]**	2.0 [−1.6, 5.6]	2.1 [−1.2, 5.5]	**4.5 [0.5, 8.4]**	2.7 [−0.9, 6.3]	**4.4 [0.4, 8.3]**
Cortisol (mcg/dL)	−1.1 [−2.5, 0.3]	0.6 [−0.9, 2.2]	−1.3 [−2.7, 0.1]	−0.8 [−2.7, 1.1]	0.4 [−1.3, 2.1]	−1.5 [−3.3, 0.4]
Cholesterol (mg/dL)	1.4 [−13.9, 16.8]	−4.0 [−21.1, 13.1]	−7.5 [−23.5, 8.5]	−10.9 [−31.1, 9.3]	−1.7 [−20.5, 17.1]	−11.0 [−31.6, 9.5]
Triglycerides (mg/dL)	24.1 [−1.6, 49.9]	1.2 [−27.4, 29.9]	17.0 [−9.7, 43.8]	17.8 [−15.8, 51.3]	9.4 [−21.8, 40.7]	9.1 [−24.9, 43.2]
High-density lipoprotein (mg/dL)	0.1 [−6.0, 6.1]	−3.2 [−10.0, 3.5]	−6.0 [−12.3, 0.3]	−4.5 [−11.9, 3.0]	−2.3 [−9.2, 4.6]	−6.0 [−13.6, 1.5]
Low-density lipoprotein (mg/dL)we	3.0 [−9.6, 15.7]	0.7 [−13.5, 14.8]	5.9 [−7.2, 19.2]	−4.3 [−21.0, 12.3]	1.4 [−14.1, 16.9]	5.9 [−11.0, 22.8]

Abbreviations: BMI, body mass index; CI, confidence interval. Bold denotes when 95% confidence intervals do not cross 0.

a For both unadjusted and adjusted models, each of the three locations was compared separately with the control region.

b Controlled for age, gender, square root of socioeconomic status, and maternal height.

## Results

A summary of the sample, anthropometry, skinfold thickness, blood pressure and blood results for exposure groups are shown in Table 1. There were 110 adolescents in the total sample, 46 (42%) of whom were girls. An additional 20 adolescents were excluded because their mothers were found to be not pregnant during the tsunami. The sample size and ages (range 12.5-13.9 years) in each exposure group were similar. The population was generally short and thin, when comparing to the WHO growth standards. The mean height-for-age and BMI-for-age z scores for the whole sample were -0.95 and -1.18 respectively. Only one child was overweight (BMI z > 2) and none were obese (BMI z > 3). Using the MPI score, the conflict and conflict-plus-tsunami groups were multidimensionally poorer than the control group, whereas the tsunami-only group was similar to the control group. Pubertal stage was more advanced in both groups of adolescents living in conflict zones compared with controls, and skinfold thickness appeared to be higher in these adolescents. However, there was no obvious difference in these outcomes among those born during the tsunami alone, compared with the control group.

Table 2 shows regression models, both unadjusted and adjusted. For all models, each of the three exposed groups was compared separately with the control group. For the univariable regression models the total sample size was 110 individuals. For multivariable regression models, this was restricted to 95 due to missing data in the maternal height variable. We consider the adjusted results to be our primary results. In these models, the trends indicate higher weight, BMI and mid-upper arm circumference in each of the three exposed groups, and higher skinfold thicknesses in the conflict and conflict-plus-tsunami group, compared with those born in the control areas. In the conflict and conflict-plus-tsunami groups, greater fatness was also evident in the adjusted models. Regarding body girths, calf circumference was greater in adolescents born in all three of the exposed groups, while hip circumference was higher in the conflict-plus-tsunami group, with a weaker trend in the same direction in the tsunami-exposed group. There was a trend toward lower waist circumference in the conflict-only group. No difference was seen in height, thigh circumference or head circumference. See Figures 2 and 3 in the appendix for height- and BMI-for-age and blood pressure differences.

Diastolic blood pressure was higher among adolescents born in the conflict and conflict-plus-tsunami zones, with a weaker trend in the tsunami group. Systolic blood pressures were also higher in the conflict and conflict-plus-tsunami groups, but all 95% confidence intervals were wider and crossed 0.

The blood results show a mixed picture. Regression analyses showed no significant differences in any of the exposed groups compared with controls, aside from a slightly higher serum insulin in the conflict and the conflict-plus-tsunami group. There was a tendency toward differences compared with controls in the adolescents born in the two conflict zone groups, with lower fasting glucose levels, HbA1C, and cholesterol, but higher triglycerides and low-density lipoproteins (Table 2).

## Discussion

In this study, we explored the association between early life exposure to two major events in Sri Lanka: the tsunami and the civil war. In adolescents born in the conflict zones, compared with the control area, we observed increased BMI, body fat, changes in body dimensions, and higher diastolic blood pressure and higher serum insulin concentration. However, no difference was found in adolescent height after adjusting for age, gender, socioeconomic status, and maternal height. In adolescents born in the tsunami regions, similar results were found for anthropometry and body circumferences, but no differences were found in skinfold thickness, blood pressure, or blood results. Our initial hypothesis of anthropometry changes exposed to maternal trauma was in part borne out in our exploratory study. While a pilot study such as this cannot give definitive results, there appeared to be some lasting effects of the conflict on adolescent anthropometry and metabolic profile, with greater trade-offs in the conflict plus tsunami areas.

The two exposures we have assessed, namely, the tsunami and the conflict, are qualitatively different in their consequences. The tsunami was a discrete event that happened at a particular time point and affected a geographical region. Its effects were devastating and it is possible that the recovery can take years, for example, fishermen losing their livelihoods, but recovery might also occur in a relatively short time.^19^ The conflict however preceded the birth of the children and continued for approximately five years after, though the effects of war can be felt many years later.^3^ In the combined-exposure area, the families experienced both acute and chronic trauma.

Our results for the conflict groups provided some support for the hypothesis that exposure to stressful events in early life, favors increased allocation of resources to “reproduction” and “defense” in adolescence.^16^ These groups had greater weight and higher markers of body fat depots that are associated with both reproduction (calf girth, hip girth, and arm skinfolds)^20,21^ and immune function (torso skinfolds).^22^ This may be related to advanced pubertal stage in the conflict region, associated with variability in body composition. Associations of prenatal stress with rapid maturation have been reported in a wide range of animal species, though the association depends on when during gestation the stress occurs.^23^

The null finding for height in the conflict groups might have arisen because faster maturation leads to earlier cessation of growth,^9^ hence these groups may yet demonstrate shorter stature at a later age when growth has ceased. The null findings for head girth are less unexpected and in keeping with our hypothesis, as head growth is generally the most canalized component of growth, and least sensitive to environmental perturbations.^13^ Overall, these results show some similarity with recent analysis of life history trade-offs among adolescent daughters in association with maternal capital, in a Brazilian birth cohort.^10^ These elevated investments in reproduction and defense were accompanied by trends toward reduced markers of homeostatic maintenance (higher DBP), which may indicate immediate costs of higher adiposity as predicted by the theory of life history trade-offs. However, the results for sons in that cohort were different, where low maternal investment was associated with early reproduction by sons, but not with elevated adiposity or cardio-metabolic risk.^11^ Therefore, the life history consequences of early exposure to trauma may differ by sex, however our study was too small to address possible sex differences. Moreover, the consequences of developmental exposure to stress may not be identical to those following developmental exposure to maternal undernutrition.

Intriguingly, some markers of noncommunicable disease (NCD) risk (blood glucose) showed trends toward lower values in those exposed to conflict. This was the opposite direction of effect to that hypothesized, which suggests that this prediction merits re-appraisal. In the short-term, it is possible that markers of circulating fuel may be lower among those prioritizing investment in adiposity, and hence indicate higher rates of lipogenesis.

The contrasting findings for conflict- and tsunami-exposed groups in skinfold thickness, blood pressure and serum insulin may relate to the duration of the exposure. Both events led to mass morbidity and loss of life, and extreme societal and economic disruption, but these events were also different. Differences in long-term health outcomes have been associated with environmental stresses in many forms and we hypothesized that these large-scale events would lead to changes in nutritional outcomes in young adolescents, with the greatest consequences in those exposed to conflict-plus-tsunami. We had assumed differences would be found in blood results beyond serum insulin. It may be that there is no true difference present or that our study was not sensitive enough to pick up differences in what are highly variable markers.

Research on the long-term consequences of early-life exposure to disasters associated with natural events has grown in recent years. Supporting our study, a systematic review from 2021 identified 37 studies that found correlations between prenatal exposure to natural disasters and birth and child health outcomes. Higher prenatal stress, in most studies measured as a change in objective psychological assessment scores, was associated with “longer gestational age, larger newborns, higher BMI and adiposity levels, and worse cognitive, motor, socio-emotional, and behavioral outcomes.” A correlation of 0.10 (95% CI 0.06, 0.15) was found between prenatal maternal stress and child BMI or adiposity. The included studies were largely from the Canada, the USA and Australia, with only four studies from low- and middle-income countries (China, Haiti, Thailand, and Vanuatu).^2^

In addition, in support of our results and hypothesis, studies of prenatal exposure to Cyclone Aila in India showed changes in skinfold thickness^24^ and also lower hand grip strength in children aged 7 to 9 years.^25^ However, contrary to our findings, in this study, the prenatally exposed group also had a smaller head circumference.^26^ War is also known to have intergenerational health effects.^3,27^ The clearest evidence comes from the Dutch Winter famine in World War II, where adults born during famines were found to have an increased risk of coronary heart disease, diabetes, obesity, and altered blood profile.^28,29^ These effects may have been evident because the follow-ups were conducted from middle-age onward, when patterns of cumulative NCD risk may manifest more strongly.

### Limitations

We acknowledge that it is difficult to account for the long-term consequences of major events, in particular a civil war. In this study, we compared children born in a war region, a tsunami region and those who were born in neither. In a retrospective study such as this, we are limited in the precision of the exposure. Ideally, we would have wanted exposure data from the time of the tsunami or during the conflict. The nature of this type of research means that these data are very difficult to collect. It is often not possible, and sometimes not ethical, to conduct research immediately postdisaster. We have therefore attempted to stratify by region and control for confounding factors that may be important.

The main limitation of this study was the sample size, meaning that we were not adequately powered and preventing us from drawing firm conclusions from the data. We could not test for possible sex differences in the associations. Given the number of outcomes, we were also at risk of multiple testing. If we correct for this, using the Benjamini-Hochberg method, the *p*-value denoting significance declines from 0.05 to 0.01. On this basis, group contrasts in anthropometry and skinfolds are still significant, whereas those for blood pressure are not (Appendix, Table 2). However, we believe this correction is overly conservative for our explorative study, and we would urge the reader to interpret the results together as composite measures of life history strategy, rather than any one result in isolation, and to treat any associations with caution. We were also unsure if pubertal stage was assessed accurately. This was done as a self-assessment with no verification, for practical reasons. Further qualitative work, with the appropriate confidentiality and ethical considerations, would help to understand pubertal self-assessment in the Sri Lankan setting.

## Conclusions and Implications

Our study showed potential anthropometric differences in children whose mothers were pregnant during the Indian Ocean tsunami and the conflict. These adolescents showed higher body fat, measured by skinfolds, than controls, with greater differences seen for those born in the conflict zones. Blood pressure and fasting insulin were also elevated in the affected groups, though the findings were less robust. The results of this type of research can help inform interventions for adolescents at risk and help to understand the full consequences of a natural disaster—both within and outside of conflict zones—that can be potentially be used in advocacy to improve services for those previously exposed.

## Supplemental Material

sj-docx-1-aph-10.1177_10105395231151730 – Supplemental material for The Long-Term Consequences of Early Life Exposure to Tsunami and Conflict on Adolescents in Sri Lanka Supplemental material, sj-docx-1-aph-10.1177_10105395231151730 for The Long-Term Consequences of Early Life Exposure to Tsunami and Conflict on Adolescents in Sri Lanka by Delan Devakumar, Laura Busert, Manoji Gitanjali Sathiadas, Pushpika Jayawardana, Angela Arulpragasam, Clive Osmond, Caroline H. D. Fall, Jonathan C. K. Wells and V. Pujitha Wickramasinghe in Asia Pacific Journal of Public Health
