# Supplementary material for: The Long-Term Consequences of Early Life Exposure to Tsunami and Conflict on Adolescents in Sri Lanka
Source: Asia Pac J Public Health. 2023 Jan 25;35(2-3):112–20. doi: 10.1177/10105395231151730 (PMC10185911; doi:10.1177/10105395231151730)
Supplement: sj-docx-1-aph-10.1177_10105395231151730 – Supplemental material for The Long-Term Consequences of Early Life Exposure to Tsunami and Conflict on Adolescents in Sri Lanka [file sj-docx-1-aph-10.1177_10105395231151730.docx]

**Appendix**

**The long-term consequences of early life exposure to tsunami and conflict on adolescents in Sri Lanka**

**Contents**

[Figure 1: Conceptual model 2](#_Toc115214016)

[Figure 2. Graph to show difference in z scores compared to control region for height- and BMI-for-age (with 95% confidence intervals) with adjusted results 3](#_Toc115214017)

[Figure 3. Graph to show difference in systolic and diastolic blood pressure compared to control region (with 95% confidence intervals) with adjusted results 4](#_Toc115214018)

[Blood sample testing procedures 5](#_Toc115214019)

[Table 1. Correlation coefficients between confounding variables and outcomes 6](#_Toc115214020)

[Table 2. Unadjusted and adjusted models showing the association (regression coefficients and 95% confidence intervals) between in-utero exposure and adolescent nutrition and blood pressure outcomes compared to the baseline (control region) with p values 7](#_Toc115214021)

## Figure 1: Conceptual model

The conceptual model was constructed by our team, based on our understanding of the literature. From this, using causal theory, we chose confounding variables to include in the analysis. Controlling for socioeconomic status and stratifying by war and tsunami in this instance would be a sufficient set of variables to control for to block all ‘backdoor paths’ between exposure and outcome. In addition to this, we added age and gender which would increase the precision of the results and considered that maternal height would also be important, given our focus on growth status of the offspring. Additional variables that may affect child growth are mediators in our conceptual model and would therefore be inappropriate to control for.

## Figure 2. Graph to show difference in z scores compared to control region for height- and BMI-for-age (with 95% confidence intervals) with adjusted results

## Figure 3. Graph to show difference in systolic and diastolic blood pressure compared to control region (with 95% confidence intervals) with adjusted results

## Blood sample testing procedures

Blood samples (6 mL) were collected in plain tubes after an overnight (12-14 hour) fast. Full blood count, fasting glucose and HBA1C were tested in the laboratory in the local teaching hospital. Plasma separation was carried out by centrifugation at 2500rpm for 10 minutes within one hour of venepuncture and samples were then frozen and transported to the University of Colombo in batches where they were tested for lipids, insulin and cortisol levels. Blood for full blood count and HbA1c was collected into an EDTA containing tube (2.5 ml) and for fasting blood sugar into sodium fluoride and potassium oxalate in 1:3 ratio containing tube. Full blood count was analysed using a fully automated analyser (Sysmex/Celdyn®). Fasting blood glucose was analysed using the GOD-PAP method (Olympus® analyser). HbA1c was analysed using HPLC (D10/Bio-Rad Variant).

Aliquots for plasma insulin, cortisol and lipid profile were stored at –20°C until analysis. Frozen samples were carefully thawed and checked for air bubbles or particulate matter prior to analysis. Assays were performed on frozen-thawed samples in batches. Quantitative determination of insulin and cortisol was carried out on a fully automated random-access LIAISON® immune-assay system (DiaSorin Spa, Germany) using Diasorin Liaison reagents as specified in the respective package inserts and Diasorin’s operating manual. Insulin levels were measured using a sandwich chemiluminescence immunoassay technique with intra assay coefficient of variation (CV) of 1.6% and inter‐assay CVs of 2.2% and cortisol levels were measured using a solid phase antigen linked technique with intra assay CV of 3.2% and inter‐assay CVs of 4.0%. Both the tests were carried out as specified in Diasorin’s operating manual, using Diasorin Liaison reagents as specified in the respective package inserts. Total cholesterol, HDL-C, LDL-C and triglyceride levels in serum were analyzed using a Cobas c111 Clinical Chemistry Analyzer (Roche Diagnostics GmbH, Germany). All these tests were enzymatic colorimetric assays and were carried out as specified in Roche’s operating manual, using Roche reagents as specified in the respective package inserts. The intra-assay CV% and Inter assay CV% for both total cholesterol and triglycerides were within 1.0%. The inter assay and intra-assay CVs for HDL-C were 1.4% and 1.6% and for LDL-C were 2.2% and 1.9% respectively.

## Table 1. Correlation coefficients between confounding variables and outcomes

|  | **Age** | **Individual Multidimensional index score** | **Maternal Height** | **Paternal Height** |
| --- | --- | --- | --- | --- |
| Height | 0.21 | -0.38 | 0.14 | -0.05 |
| Weight | 0.12 | -0.46 | 0.12 | -0.09 |
| HAZ | 0.08 | -0.37 | 0.16 | -0.03 |
| BMIZ | -0.03 | -0.45 | 0.05 | -0.14 |
| Head circumference | 0.12 | -0.12 | 0.12 | -0.093 |
| Mid-upper arm circumference | 0.13 | -0.39 | 0.17 | -0.07 |
| Waist circumference | 0.26 | -0.40 | 0.11 | -0.01 |
| Thigh circumference | 0.15 | -0.51 | 0.20 | -0.040 |
| Hip circumference | -0.06 | -0.28 | 0.22 | -0.07 |
| Calf circumference | -0.21 | 0.10 | 0.17 | -0.001 |
| Biceps Skinfold thickness | 0.08 | 0.02 | 0.12 | -0.16 |
| Triceps Skinfold thickness | 0.08 | 0.06 | 0.07 | -0.22 |
| Subscapular Skinfold thickness | 0.04 | -0.03 | 0.01 | -0.19 |
| Suprailiac Skinfold thickness | -0.02 | 0.03 | 0.03 | -0.17 |
| Systolic Blood pressure | 0.16 | -0.05 | -0.04 | -0.14 |
| Diastolic Blood pressure | -0.09 | -0.01 | 0.01 | 0.11 |

## Table 2. Unadjusted and adjusted models showing the association (regression coefficients and 95% confidence intervals) between in-utero exposure and adolescent nutrition and blood pressure outcomes compared to the baseline (control region) with p values

|  | **UNADJUSTED** | | |
| --- | --- | --- | --- |
|  | **Conflict**  (95% CI) | **Tsunami**  (95% CI) | **Conflict plus Tsunami**  (95% CI) |
| Height (cm) | -5.6 (-9.6, -1.7) p = 0.006 | -3.5 (-7.9, 1.0) p = 0.123 | -4.8 (-8.9, -0.6) p = 0.024 |
| Weight (kg) | -2.68 (-7.36, 1.99) p = 0.257 | 0.88 (-4.32, 6.09) p = 0.737 | -1.17 (-6.03, 3.70) p = 0.636 |
| Height for age | -0.7 (-1.2, -0.2) p = 0.010 | -0.2 (-0.8, 0.4) p = 0.476 | -0.5 (-1.0, 0.1) p = 0.098 |
| BMI for age | -0.2 (-0.1, 0.7) p = 0.711 | 0.4 (-0.5, 1.3) p = 0.374 | 0.2 (-0.7, 1.0) p = 0.699 |
| Head circumference (cm) | -0.3 (-1.1, 0.6) p = 0.575 | 0.1 (-0.9, 1.0) p = 0.874 | -0.3 (-1.2, 0.6) p = 0.447 |
| Mid-upper arm circumference (cm) | -0.8 (-2.5, 0.8) p = 0.323 | 0.3 (-1.6, 2.1) p = 0.760 | 0.1 (-1.7, 1.8) p = 0.927 |
| Waist circumference (cm) | -8.8 (-12.6, -5.0) p = <0.001 | -5.4 (-9.6, -1.1) p = 0.014 | -5.4 (-9.4, -1.4) p = 0.009 |
| Hip circumference (cm) | -2.2 (-6.7, 2.4) p = 0.351 | 3.5 (-1.6, 8.6) p = 0.178 | 3.5 (-1.2, 8.3) p = 0.144 |
| Thigh circumference (cm) | -5.9 (-8.7, -3.0) p = <0.001 | -0.6 (-3.8, 2.6) p = 0.705 | -3.4 (-6.3, -0.4) p = 0.026 |
| Calf circumference (cm) | 6.7 (4.8, 8.6) p = <0.001 | 8.4 (6.2, 10.5) p = <0.001 | 7.9 (5.9, 9.9) p = <0.001 |
| **Skinfold thickness** |  |  |  |
| Biceps (mm) | 1.1 (-1.0, 3.2) p = 0.293 | -0.1 (-2.5, 2.2) p = 0.915 | 3.9 (1.7, 6.1) p = 0.001 |
| Triceps (mm) | 1.2 (-1.1, 3.5) p = 0.301 | -0.3 (-2.9, 2.2) p = 0.798 | 4.0 (1.6, 6.4) p = 0.001 |
| Subscapular (mm) | 1.0 (-1.3, 3.2) p = 0.404 | -1.8 (-4.3, 0.7) p = 0.156 | 3.0 (0.7, 5.4) p = 0.012 |
| Suprailiac (mm) | 3.0 (0.0, 5.9) p = 0.047 | -2.0 (-5.2, 1.3) p = 0.230 | 4.6 (1.6, 7.7) p = 0.003 |
| **Blood pressure** |  |  |  |
| Systolic blood pressure (mmHg) | 3.6 (-2.0, 9.1) p = 0.203 | -1.4 (-7.5, 4.8) p = 0.663 | -1.3 (-7.1, 4.5) p = 0.652 |
| Diastolic blood pressure (mmHg) | 4.3 (0.5, 8.1) p = 0.028 | 2.8 (-1.5, 7.0) p = 0.198 | 4.8 (0.9, 8.8) p = 0.017 |
| **Blood results** |  |  |  |
| Fasting glucose (mg/DL) | -4.2 (-8.9, 0.4) p = 0.075 | -0.2 (-5.4, 5.0) p = 0.933 | -9.4 (-14.2, -4.5) p = <0.001 |
| HBA1C (%) | -0.3 (-0.5, -0.0) p = 0.029 | 0.1 (-0.1, 0.4) p = 0.377 | -0.4 (-0.6, -0.1) p = 0.003 |
| Serum insulin (mIU/L) | 3.6 (0.3, 6.8) p = 0.030 | 2.0 (-1.6, 5.6) p = 0.281 | 2.1 (-1.2, 5.5) p = 0.204 |
| Cortisol (mcg/dl) | -1.1 (-2.5, 0.3) p = 0.115 | 0.6 (-0.9, 2.2) p = 0.429 | -1.3 (-2.7, 0.1) p = 0.068 |
| Cholesterol (mg/dL) | 1.4 (-13.9, 16.8) p = 0.854 | -4.0 (-21.1, 13.1) p = 0.647 | -7.5 (-23.5, 8.5) p = 0.356 |
| Triglycerides (mg/dL) | 24.1 (-1.6, 49.9) p = 0.066 | 1.2 (-27.4, 29.9) p = 0.933 | 17.0 (-9.7, 43.8) p = 0.210 |
| High-density lipoprotein (mg/dL) | 0.1 (-6.0, 6.1) p = 0.981 | -3.2 (-10.0, 3.5) p = 0.346 | -6.0 (-12.3, 0.3) p = 0.062 |
| Low-density lipoprotein (mg/dL) | 3.0 (-9.6, 15.7) p = 0.635 | 0.7 (-13.5, 14.8) p = 0.927 | 5.9 (-7.2, 19.2) p = 0.374 |

*Controlled for age, gender, square root of socioeconomic status and maternal height

|  | **ADJUSTED*** | | |
| --- | --- | --- | --- |
|  | **Conflict**  (95% CI) | **Tsunami**  (95% CI) | **Conflict plus Tsunami**  (95% CI) |
| Height (cm) | 0.7 (-4.0, 5.5) p = 0.757 | -0.1 (-4.5, 4.4) p = 0.975 | 1.1 (-3.6, 6.0) p = 0.627 |
| Weight (kg) | 6.85 (1.45, 12.25) p = 0.014 | 5.95 (0.93, 10.97) p = 0.021 | 9.30 (3.82, 14.78) p = 0.001 |
| Height for age | 0.1 (-0.5, 0.8) p = 0.670 | 0.0 (-0.6, 0.6) p = 0.975 | 0.2 (-0.4, 0.8) p = 0.559 |
| BMI for age | 1.3 (0.4, 2.2) p = 0.007 | 1.0 (0.2, 1.9) p = 0.015 | 2.0 (1.1, 2.9) p = <0.001 |
| Head circumference (cm) | 0.3 (-0.9, 1.5) p = 0.614 | 0.4 (-0.7, 1.5) p = 0.448 | 0.3 (-0.9, 1.5) p = 0.600 |
| Mid-upper arm circumference (cm) | 2.1 (0.1, 4.0) p = 0.035 | 1.9 (0.1, 3.7) p = 0.042 | 3.4 (1.5, 5.4) p = 0.001 |
| Waist circumference (cm) | -4.4 (-9.3, 0.5) p = 0.078 | -2.7 (-7.3, 1.8) p = 0.235 | 0.1 (-4.9, 5.0) p = 0.979 |
| Hip circumference (cm) | 4.3 (-1.4, 9.9) p = 0.135 | 5.1 (-0.2, 10.3) p = 0.057 | 9.3 (3.5, 15.0) p = 0.002 |
| Thigh circumference (cm) | -0.9 (-4.4, 2.5) p = 0.588 | 1.9 (-1.3, 4.0) p = 0.244 | 2.0 (-1.5, 5.5) p = 0.256 |
| Calf circumference (cm) | 7.9 (5.3, 10.5) p = <0.001 | 9.3 (6.9, 11.7) p = <0.001 | 10.0 (7.4, 12.6) p = <0.001 |
| **Skinfold thickness** |  |  |  |
| Biceps (mm) | 3.5 (0.9, 6.3) p = 0.010 | 0.5 (-2.0, 3.1) p = 0.678 | 6.0 (3.2, 8.7) p = <0.001 |
| Triceps (mm) | 3.1 (0.3, 5.9) p = 0.028 | 0.2 (-2.4, 2.9) p = 0.871 | 5.7 (2.9, 8.5) p = <0.001 |
| Subscapular (mm) | 3.6 (0.9, 6.4) p = 0.010 | -1.0 (-3.6, 1.5) p = 0.433 | 5.2 (2.4, 8.0) p = <0.001 |
| Suprailiac (mm) | 5.1 (1.7, 8.6) p = 0.004 | -1.5 (-4.7, 1.8) p = 0.372 | 7.3 (3.8, 10.8) p = <0.001 |
| **Blood pressure** |  |  |  |
| Systolic blood pressure (mmHg) | 6.3 (-1.4, 13.9) p = 0.107 | 1.7 (-5.4, 8.8) p = 0.633 | 3.2 (-4.6, 10.9) p = 0.422 |
| Diastolic blood pressure (mmHg) | 5.6 (0.5, 10.7) p = 0.031 | 4.2 (-0.5, 9.0) p = 0.077 | 6.7 (1.5, 11.8) p = 0.012 |
| **Blood results** |  |  |  |
| Fasting glucose (mg/DL) | -5.2 (-10.9, 0.5) p = 0.072 | 3.6 (-1.7, 8.9) p = 0.178 | -5.3 (-11.1, 0.4) p = 0.070 |
| HBA1C (%) | -0.3 (-0.6, 0.1) p = 0.101 | 0.1 (-0.2, 0.5) p = 0.353 | -0.3 (-0.7, 0.0) p = 0.057 |
| Serum insulin (mIU/L) | 4.5 (0.5, 8.4) p = 0.027 | 2.7 (-0.9, 6.3) p = 0.138 | 4.4 (0.4, 8.3) p = 0.031 |
| Cortisol (mcg/dl) | -0.8 (-2.7, 1.1) p = 0.389 | 0.4 (-1.3, 2.1) p = 0.618 | -1.5 (-3.3, 0.4) p = 0.125 |
| Cholesterol (mg/dL) | -10.9 (-31.1, 9.3) p = 0.288 | -1.7 (-20.5, 17.1) p = 0.857 | -11.0 (-31.6, 9.5) p = 0.288 |
| Triglycerides (mg/dL) | 17.8 (-15.8, 51.3) p = 0.296 | 9.4 (-21.8, 40.7) p = 0.549 | 9.1 (-24.9, 43.2) p = 0.595 |
| High-density lipoprotein (mg/dL) | -4.5 (-11.9, 3.0) p = 0.235 | -2.3 (-9.2, 4.6) p = 0.507 | -6.0 (-13.6, 1.5) p = 0.116 |
| Low-density lipoprotein (mg/dL) | -4.3 (-21.0, 12.3) p = 0.608 | 1.4 (-14.1, 16.9) p = 0.856 | 5.9 (-11.0, 22.8) p = 0.491 |
